# Molecular Markers: A New Paradigm in the Prediction of Sperm Freezability

**DOI:** 10.3390/ijms24043379

**Published:** 2023-02-08

**Authors:** Michal Ďuračka, Filip Benko, Eva Tvrdá

**Affiliations:** 1 AgroBioTech Research Centre, Slovak University of Agriculture in Nitra, Tr. A. Hlinku 2, 949 76 Nitra, Slovakia; 2 Institute of Applied Biology, Faculty of Biotechnology and Food Sciences, Slovak University of Agriculture in Nitra, Tr. A. Hlinku 2, 949 76 Nitra, Slovakia; 3 Institute of Biotechnology, Faculty of Biotechnology and Food Sciences, Slovak University of Agriculture in Nitra, Tr. A. Hlinku 2, 949 76 Nitra, Slovakia

**Keywords:** spermatozoa, cryopreservation, markers, genomics, epigenetics, proteomics, oxidative stress, freezability

## Abstract

For decades now, sperm cryopreservation has been a pillar of assisted reproduction in animals as well as humans. Nevertheless, the success of cryopreservation varies across species, seasons, and latitudes and even within the same individual. With the dawn of progressive analytical techniques in the field of genomics, proteomics, and metabolomics, new options for a more accurate semen quality assessment have become available. This review summarizes currently available information on specific molecular characteristics of spermatozoa that could predict their cryotolerance before the freezing process. Understanding the changes in sperm biology as a result of their exposure to low temperatures may contribute to the development and implementation of appropriate measures to assure high post-thaw sperm quality. Furthermore, an early prediction of cryotolerance or cryosensitivity may lead to the establishment of customized protocols interconnecting adequate sperm processing procedures, freezing techniques, and cryosupplements that are most feasible for the individual needs of the ejaculate.

## 1. Introduction

**Figure 1:**
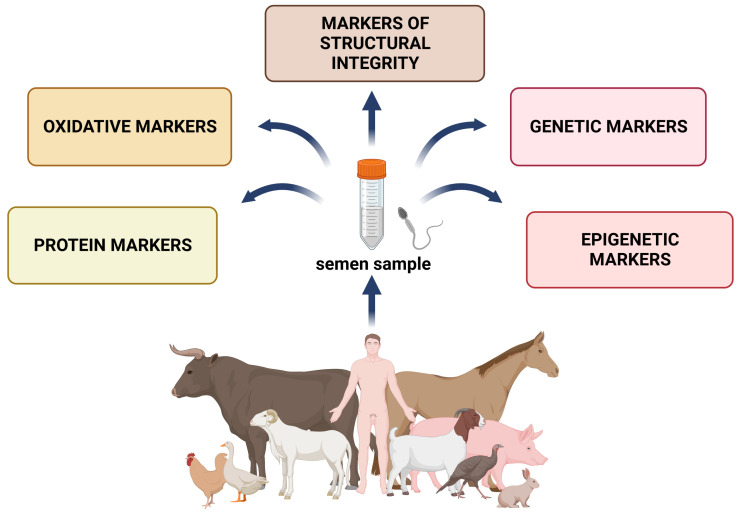
Conventional and modern markers suitable for the prediction of sperm freezability. Created with www.BioRender.com (supplementary S1: Confirmation of Publication and Licensing Rights) (accessed on 30 December 2022).

The history of semen preservation dates back to the 18th century when Lazaro Spallanzani (1776) experimented with snow-cooled human spermatozoa [1]. The potential to freeze spermatozoa in order to preserve their viability has been known for almost 70 years, while the discovery of glycerol as a cryoprotectant brought substantial achievements in the field [2]. The first successfully born offspring in the 1950s (cow, human, pig, and horse) and 1960s (sheep) allowed the emergence of sperm cryobanks [3]. Nowadays, freezing protocols for cryopreserved semen enable a desirable post-thawed recovery of sperm viability in almost every livestock species as well as humans. Every critical point of the semen cryopreservation technology has improved with time, including the cooling and freezing period, temperatures, and composition of the semen extender. In humans, cryopreservation offers reproductive management for patients receiving radiotherapy, chemotherapy, or reproductive surgery with testicular or ejaculatory disorders as a side effect [4]. In animal species, cryopreservation represents an important technology that allows genetic selection and improves the success rate of artificial insemination and preservation of endangered breeds in cryobanks. Independent of the procedure selected to preserve spermatozoa at low temperatures, the most important factor contributing to the resulting sperm vitality and fertilization ability is the semen quality prior to the freezing process and the changes male gametes must withstand during their exposure to low temperatures. Unfortunately, the full potential of semen cryopreservation is not fully exploited yet, since a complete understanding of the sperm physiology prior to or during the freezing and thawing process mandatory to ensure maximal success is still lacking. As such, this review summarizes the current understanding of the markers predicting sensitivity to cryopreservation, or factors that may be helpful in evaluating sperm cryoresistance, and offers strategies for improving the freezability of individuals considered “poor freezers” (Figure 1).

## 2. Markers of Structural Integrity

Motility evaluation seems to be a cardinal parameter in the assessment of spermatozoa quality. Even though computer-assisted semen analysis (CASA) brings objectification of motility measurement, manual counting of moving/nonmoving cells is still the most common technique used in the practice. Besides counting sperm motility and concentration, CASA analysis also allows studying sperm trajectory expressed through curvilinear velocity (VCL), straight-line velocity (VSL), and average velocity path. A significant reduction in sperm motility is, in most cases, a critical side effect of long-term preservability of human [5], bull [6], or fish semen [7]. However, there are several animal species including boars, stallions, rams, camels, bucks, and avian species where cryopreservation is less often used because of a notable variability of post-thaw sperm survival rate [8]. Despite cryopreservation protocols being well optimized, post-thaw sperm motility is significantly decreased in every species. However, a recent study [5] on human semen revealed that sperm motility significantly increases with the lasting time of thawing. The authors evaluated sperm motility 20 min and 40 min after thawing. The motility was significantly increased after 40 min of thawing. Another study by Schäfer-Somi and Tichy [9] found overall sperm behavior as a good predictor of freezability. High specificity and accuracy were reported when combining several motility parameters including progressive motility, VCL, straightness, and linearity (LIN) [9]. Davis et al. [10] used a complex iterative multiple regression method that had been established to predict cryoresistance in human spermatozoa. As VSL and LIN were the most commonly predictive primary variables, the difference in VSL between the subpopulation with the highest rates and the subpopulation with the lowest rates was used to predict post-thaw sperm viability. Núñez-Martínez et al. [11] hypothesized that the higher the sperm motility parameters VSL and VCL in fresh canine semen, the higher the resulting fertilizing potential after thawing.

Several ultrastructural reports have been published to assess the disintegration of the plasma membrane and its subsequent dysfunction during cryopreservation [12,13,14]. The membrane destabilization is caused by thermal and osmotic factors. In brief, the transition to colder or warmer temperatures and the local exposition to water or high concentrations of salts can change the membrane organization and thereby account for the loss of selective permeability [15]. Maintenance of the physical properties of the membranes could contribute to increased cryotolerance of spermatozoa. The hypo-osmotic swelling (HOS) test reflects on the functional acrosome integrity and integrity of the tail region in a hypo-osmotic environment [16]. Even though eosin-nigrosin (EN) staining brings reliable results when identifying fresh semen quality, two studies [17,18] found this technique inappropriate when evaluating post-thaw spermatozoa. On the contrary, Trypan-blue/Giemsa (TBG) staining belongs to bright-field staining techniques with good credibility when analyzing the acrosomal damage in cryopreserved spermatozoa [19]. The acrosome intactness test (AIT) introduced by Gopalkrishnan et al. [20] belongs to simple but tedious tests based on the halo effect of intact acrosomes in samples diluted with PBS and glucose solution smeared on gelatin-coated slides. Although bright-field microscopy offers some straightforward techniques to evaluate the sperm membrane, flow cytometry combined with fluorescent stains is extensively used. Fluorescent dyes are grouped based on their permeability. Some of them directly pass through the membrane in the case of a damaged membrane and are observable under the microscope. Other dyes acquire fluorescence while passing through the intact plasma membrane. More accurate results are achieved in the case of double- or triple-staining protocols. Carboxyfluorescein diacetate (CFDA) is frequently used as an indicator of an intact membrane and is usually combined with propidium iodide (PI), permeable only for necrotic cells [21]. According to Watts [22], CFDA/PI staining is more dependable than the EN technique because at <80% of membrane-intact spermatozoa, EN overestimates the percentage of intact cells. Except for live and dead fluorescent staining, another view on the sperm viability rates may be provided by apoptotic dyes such as Annexin-V, YO-PRO-1, fluorescent inhibitor of caspases (FLICA), and Caspases 3/7. The acrosome status of post-thawed as well as pre-thawed spermatozoa has been monitored using peanut agglutinin (PNA), fluorescein isothiocyanate (FITC) conjugate [23], or, alternatively, by *Pisum sativum* agglutinin (PSA) [24]. However, PNA agglutinin binding to β-galactose moieties on the acrosome surface showed to be less nonspecific opposite to PSA agglutinin, which binds to α-mannose and α-galactose moieties inside the acrosome matrix [25]. Additionally, triple staining was used to predict the sustainability of cryopreservation: YO-PRO-1 and SNARF-1 correlate with sperm cryosurvival while ethidium homodimer-stained spermatozoa correlate negatively with post-thaw survival [26]. Similar results were observed using Annexin-V/FITC/PI triple staining and phycoerythrin-conjugated PNA (PE-PNA), SYBR-14, and PI, which was utilized to effectively assess the plasma and acrosome membrane [27,28]. The chlortetracycline (CTC) assay belongs to calcium-binding assays. Three basic patterns are recognized: noncapacitated spermatozoa, where the whole sperm head is CTC-positive; capacitated spermatozoa, where the sperm head is stained excluding the postacrosomal region; and acrosome-reacted spermatozoa, which are characterized by a bright fluorescent band in the equatorial segment [21]. Another study considered the double Hoechst 33258/CTC staining assay as a good predictor of male fertility in various breeds [29]. Nevertheless, Ryu et al. [30] reported no significant differences in the percentage of live capacitated, noncapacitated, and acrosome-reacted spermatozoa amongst high freezing-tolerant and low freezing-tolerant spermatozoa. In contrast, other studies [21,31,32] demonstrated changes in the capacitation status of freeze–thawed spermatozoa. On the other hand, mitochondrial membrane potential (MMP) showed significant differences between sperm donors with good and bad freezability. Moreover, MMP correlates with sperm motility and viability. Yeste et al. [33] reported that MMP could be considered a good cryotolerance marker in stallion spermatozoa. However, the authors justified the difference between good and bad freezability also by differences in reactive oxygen species (ROS) levels between the studied groups. MMP was revealed as one of the crucial markers of post-thaw sperm quality because of ATP production and sperm motility. Recent studies showed that mitochondrial-targeted treatment before cryopreservation significantly improves post-thawed sperm quality [34]. There are several fluorescent dyes such as Rhodamine 123, 5,5′,6,6′-tetrachloro-1,1′,3,3′ tetraethylbenzymidazolyl carbocyanine iodine (JC-1), carbocyanine derivative 3,3′-dihexyloxacarbocyanine iodide (DiOC_6_), Chloromethyl-X-rosamine (CMX-Ros), tetramethylrhodamine ethyl ester (TMRE), and MitoTracker Green [35]. Even though the JC-1 (5,5′,6,6′-tetrachloro-1,1′,3,3′-tetraethylbenzimidazolylcarbocyanine iodide) dye shows the best specificity amongst the tested stains, other dyes were particularly suitable for multiparametric staining [36]. Although Alamo et al. [37] showed that MMP may serve as a reliable predictor of sperm motility, some reports argue that traditional markers such as MMP or ATP levels are not able to accurately distinguish poor and good freezability of boar semen. Therefore, there is a need for new markers that may emerge from metabolomic studies [33,38].

**Figure 2:**
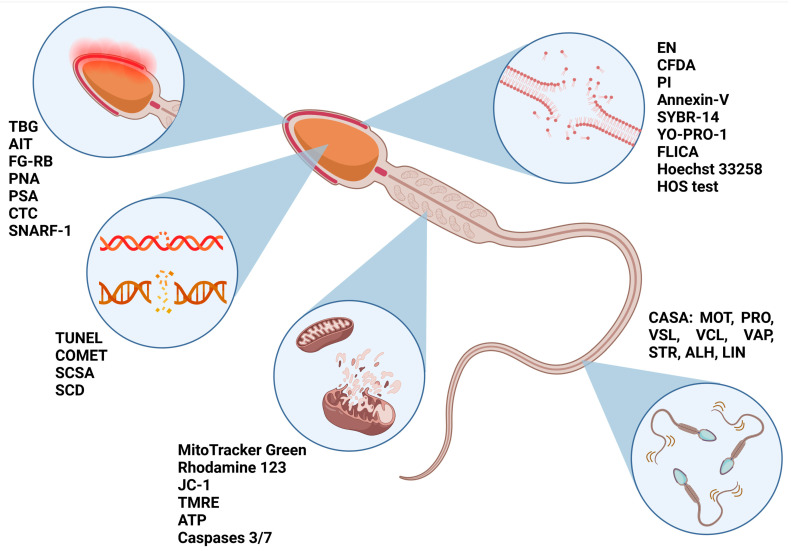
Markers allowing cryotolerance predictivity of spermatozoa before freezing. TBG—trypan blue/Giemsa staining; AIT—acrosome intactness test; FG-RB—fast green/rose Bengal staining; PNA—peanut agglutinin; PSA—*Pisum sativum* agglutinin; CTC—chlortetracycline; SNARF-1—Seminaphtharhodafluor 1; TUNEL—Terminal deoxynucleotidyl transferase dUTP Nick-End Labeling; Comet assay—single-cell gel electrophoresis; SCD—sperm chromatin dispersion test; JC-1—5,5′,6,6′-tetrachloro-1,1′,3,3′ tetraethylbenzymidazolyl carbocyanine iodine; TMRE—tetramethylrhodamine ethyl ester; ATP—adenosine triphosphate; EN—eosin-nigrosin staining; CFDA—carboxyfluorescein diacetate; PI—propidium iodide; FLICA—fluorochrome-labeled inhibitors of caspases; HOS—hypo-osmotic swelling test; CASA—computer-assisted sperm analysis; MOT—total motility; PRO—progressive motility; VSL—straight-line velocity; VCL—curvilinear velocity; VAP—average path velocity; STR—straightness; ALH—amplitude of lateral head displacement; LIN—linearity. Created with www.BioRender.com (supplementary S2: Confirmation of Publication and Licensing Rights) (accessed on 5 February 2023).

Whereas an intact structure of membranes and functional sperm kinematics form a prerequisite for successful post-thaw fertilization, an intact DNA molecule is a key factor in the prognosis of fertility. Therefore, the difference between sperm DNA structure before and after freezing could suggest the suitability of a semen sample for eventual cryopreservation. DNA fragmentation was revealed as a species-selective marker of cryotolerance predictivity related to the cysteine residues in protamine 1 as well as an unstable protamine ratio [39]. Directly, DNA fragmentation can be analyzed using the TdT-mediated dUTP-biotin nick end labeling (TUNEL) or Comet assay (single-cell gel electrophoresis assay). Indirect methods such as the sperm chromatin structure assay (SCSA) and sperm chromatin dispersion (SCD) were significantly correlated with direct methods in human and some animal species. However, in pigs, indirect methods showed less reliable results, which could be caused by their highly impermeable and difficult-to-decondense chromatin [40]. Nevertheless, the SCSA assay remains the easiest, fastest, and most cost-effective method for the evaluation of DNA damage in spermatozoa [41]. Yeste et al. [42] suggested that semen freezability is associated with nucleoprotein structure. In particular, disulfide bonds between two nucleoproteins are highly susceptible to freezing and thawing. It seems that good freezers are able to better counteract temperature changes, while a smaller resistance of disulfide bonds could be explained by defective spermatogenesis or epididymal maturation. On the other hand, higher amounts of heat shock protein 90 would provide higher protection against temperature changes in good freezers. Therefore, differences in the protein expression could bring more reliable information on cryotolerance predictability. At the same time, structural and oxidative markers allow for defining cell function in the first place. This function is defined by a series of exogenous variables that are mainly related to the in vitro manipulation of the semen specimen. For example, cell viability is largely mediated by the behavior of one molecular marker or another; therefore, the sperm functional activity following the freeze–thaw process is conditioned by their presence or absence. A summary of structural biomarkers previously related to cryotolerance/cryosensitivity is displayed in Figure 2.

## 3. Oxidative Markers

Oxidative stress is a major factor responsible for poor post-thaw spermatozoa quality. Weak antioxidant protection of sperm cells allows rising ROS levels to result in oxidative stress. In particular, superoxide radicals are generated during the cryopreservation and thawing process. The formation of free radicals leads to a chain reaction of oxidative insults starting with lipid peroxidation. However, every cell component including proteins, nucleic acids, and sugars is vulnerable to oxidative insults. A study published by Jakop et al. [43] showed that detached acrosomes characteristic of spermatozoa from “poor freezer” bulls are a consequence of changes in the lipid composition of the sperm membranes and altered antioxidant capacity of the seminal fluid. Malondialdehyde (MDA) levels, as a consequence of lipid peroxidation (LPO), were shown to be significantly lower in good freezers when compared to poor freezers [44]. The abundance of polyunsaturated fatty acids (PUFAs) in sperm membranes as well as seminal fluid [44,45] were also significantly correlated with cryoresistance. Likewise, an inverse association was found for monounsaturated fatty acid (MUFA) concentration. Their higher content in fresh seminal plasma led to decreased sperm motility and viability after thawing, indicating that the fatty acid composition could predict the freezability of spermatozoa and their post-thawed quality. Direct associations were shown between the total antioxidant capacity (TAC) of the seminal plasma and motility markers such as VAP and VSL, meaning that fresh ejaculates with a higher TAC are more appropriate for cryopreservation [45]. Vigolo et al. [46] studied relationships between oxidative markers in the seminal plasma and sperm motility properties in bovine semen before and after cryopreservation. In this study, advanced oxidative protein products (AOPPs) and thiol concentrations were significantly higher in semen with high motility than in low-sperm-motility samples. However, no significant difference was observed between the groups after thawing. Additionally, a different study [47] revealed a positive correlation between AOPPs and thiol concentrations and the kinetic properties of spermatozoa in fresh semen, while the authors emphasized a positive relation to semen freezability. Higher AOPPs and thiol concentrations resulted in higher total motility, progressive motility, and linearity of spermatozoa after thawing. Therefore, further analyses of seminal metabolites in relation to the prediction of sperm freezability are needed. Catalán et al. [48] studied relationships between seminal plasma antioxidants and sperm cryotolerance in stallion semen. Based on the post-thaw motility and SYBR-14^+^/PI^-^ rates, the ejaculates were divided into good and poor freezability groups. Their results showed that mostly enzymatic antioxidants paraoxonase type 1 (PON1) and superoxide dismutase (SOD) and nonenzymatic TAC positively correlated with good freezability, while the ferrous-to-ferric-based oxidative stress index (OSI) positively correlated with poor freezability of spermatozoa. On the contrary, intracellular superoxide was found to be higher in poor-freezability semen when compared to good-freezability ejaculates. Another study [49] recorded a good predictive value for post-thaw recovery when analyzing SOD activity, TAC, the ferric-reducing ability of plasma (FRAP), PON1, and glutathione peroxidase 5 (GPx-5). The abovementioned reports have shown that the prediction of cryotolerance in semen is more likely based on antioxidant rather than pro-oxidative markers. In other words, we may assume that markers of poor freezers are more related to lower antioxidant activity in comparison to markers of oxidative damage.

## 4. Genetic Markers

Despite the popular opinion that mature spermatozoa are transcriptionally silent, a complex of RNA transcripts was identified and associated with motility, capacitation, condensation of chromatin, and fertility potential as well as early embryo development. Some studies suggest that these RNAs are only remains trapped in a reduced cytoplasmic content and represent just past events of spermatogenesis. However, reverse transcriptases in mature cells remain active, which may activate the translation of stored sperm-associated mRNAs mainly localized in the head, but a limited number of mRNAs is also localized in the midpiece region and flagellar fibrous sheath [50,51,52].

Through the years, a wide spectrum of mammalian sperm-associated mRNAs and noncoding RNAs were identified using microarrays and RT-PCR including ribosomal, mitochondrial, microRNAs (small noncoding RNAs of about 18–24 nucleotides), piwi-interacting RNAs, and small interfering RNAs. These fragments of RNA play important roles in the monitoring of the historical progress of spermatogenesis and cell response to environmental changes induced by cryopreservation. It was confirmed that cryopreservation procedures including vitrification or freezing/thawing significantly contribute to the degradation of mRNA and a number of target transcripts, which may be used as a potential genetic marker (Table 1) of sperm quality and fertility [53,54]. Recently, many studies collected data about the precise biological functions of mRNAs and changes in transcript levels of sperm-related selected genes during cryopreservation for the improvement and optimization of these procedures in different animal species.

Nazari et al. [55] studied the impact of cryopreservation on the motility and gene expression of testis-specific serine kinase 6 (TSSK6) and both protamine 1 (PRM1) and 2 (PRM2) in epididymal bovine spermatozoa. As a part of AMP-activated protein kinases, TSSK6 is postmeiotically expressed in spermatids and mature cells, which is associated with a high motility status and late gamete fusion, while PRMs are related to protamine/histone exchange, initiation of transcription, and DNA methylation. Due to freezing/thawing, decreased levels of TSSK6, PRM1, and PRM2 encoding transcripts were present in cells with slow progressive motility in comparison to fresh samples where the relative expression was higher. Moreover, the family of TSSKs had the ability to interact with heat shock proteins (HSPs), which worked as a regulator of their levels in the germ cells and provided them protection against protein instability, ubiquitination, and proteasome-dependent degradation [55,65,66]. 

In the case of the high motility status of frozen–thawed bull spermatozoa, a group of full-length transcripts encoding genes related to capacitation and fertilization such as PLCZ1 (phospholipase C zeta 1), CRISP2 (cysteine-rich secretory protein 2), CLGN1 (calmegin 1), and ADAM5P (ADAM metalloproteinase domain 5) was detected using RNA-Seq [56]. 

As a cellular response to cold shock and oxidative damage, a significant upregulation of differentially expressed genes in RPL31 (ribosomal protein L31), GCLC (glutamate cysteine ligase catalytic subunit), ROS regulator CYB5R4 (cytochrome b5 reductase 4), and CCT5 (chaperonin containing TCP1 subunit 5), which is responsible for proper folding of cytoskeletal proteins, was observed between fresh and cryopreserved bovine spermatozoa [57,58,59]. According to Card et al. [60] downregulation of a transcript, which coded COX7C (cytochrome oxidase subunit 7C), is responsible for the disruption of the mitochondrial metabolism and electron transport chain in low-fertility bulls associated with the freezing/thawing procedure.

Pang et al. [61] suggested that the HSPD1 (heat shock protein family D member 1) gene, which coded the HSPD1 protein, can be used as an indicator of male fertility because levels of mRNA expression positively correlated with the level of sperm motility and motion kinematics, which supports mutual spermatozoa/oocyte receptor-mediated interactions and increases the litter size of boars [61]. A potential link between post-thaw quality and good or poor freezability of boar spermatozoa was also evaluated through the expression of three selected stress-related genes TXNRD1 (thioredoxin reductase 1), HSPA4L (heat shock protein family A member 4), and ATP1B1 (sodium/potassium-transporting ATPase subunit beta-1). Relative mRNA expressions of TXNRD1 as well as HSPA4L were significantly higher in the frozen samples of the poor-semen group, which may be associated with increased oxidative damage and stress conditions. However, TXNRD1 should work as a scavenger of ROS by controlling the redox state of oxidized proteins; however, even when the expression was higher in the cryopreserved group, the potential cryo-induced oxidative damage may have decreased the quality of frozen boar spermatozoa after thawing. The ATP1B1 gene may be used as a cryotolerance marker of spermatozoa to cold shock because a significant loss of mRNA expression as well as ATP1B1 protein in fresh prefreeze samples predicts reduced freezability [62]. 

Another promising approach for early detection of sperm freezability is through single nucleotide polymorphisms (SNPs) of the stress-related gene STK35 (serine/threonine kinase 35) expressed in the testis, motility-related gene IFT27 (intraflagellar transport protein 27) localized in the sperm midpiece region, or genes linked with reduced cryo-inflicted lipid peroxidation such as MAP3K20 (mitogen-activated protein kinase 20), MS4A2 (membrane spanning 4-domains A2), and ROBO1 (roundabout guidance receptor 1). The STK35 and IFT27 genes showed the presence of SNP in promoters, which may regulate their transcriptional activity and binding of transcription factors [67,68]. 

Similarly, using transcriptome RNA-seq, a spectrum of sperm-associated gene transcripts such as FOS (FOS gene family), NFATC3 (nuclear factor of activated T cells 3), EAF2 (ELL associated factor 2), BAMBI (BMP and activin membrane-bound inhibitor), PTPRU (protein tyrosine phosphatase receptor type U), PTPN2 (protein tyrosine phosphatase nonreceptor type 2), MT-ND6 (mitochondrially encoded NADH ubiquinone oxidoreductase core subunit 6), and ACADM (acyl-CoA dehydrogenase medium chain) was mainly detected in poor-freezability ejaculates of Polish large white boars, which can be an indicator of a decreased cryotolerance [52].

In humans, by using microarrays, significant differences were observed in the expression of several genes such as TPX-1 (testis-specific protein 1), LDHC (lactate dehydrogenase C), AKAP4 (A-kinase anchoring protein 4), HSBP1 (heat shock binding protein 1), and CLU (clusterin), which were overexpressed in samples with a low DNA fragmentation index [63]. Qin et al. [64] suggest that ACO2 (aconitate 2) present in the midpiece region and PKM2 (pyruvate kinase M2) localized in the body and tail of spermatozoa can be used as predictors of human sperm freezability. The expression of both genes was significantly higher in the good-freezability ejaculates against the poor ones [64]. A summary of the most studied genetic markers associated with sperm freezability is provided in Table 1.

## 5. Epigenetic Markers

It has been previously confirmed that the cryopreservation procedure leads to temperature and osmotic changes, which may induce transcriptomic and proteomic molecular disturbances in spermatozoa [69,70]. From the epigenetic point of view, cryopreservation is also responsible for the profile modification of specific genes, which ensures most sperm functions such as motility, acrosome reaction, capacitation, and fusion with the oocyte [71]. Epigenetic modifications regulate gene expression by the transformation of chromatin structure and changes in DNA availability. In general, epigenetic alternations of DNA molecules include chromatin remodeling, DNA methylation, post-translational modifications of histones, and regulation of gene expression through to miRNA [72,73]. 

One of the most common epigenetic mechanisms is DNA methylation, which takes place on the cytosine-phosphate-guanine (CpG) via methylation of the C5 position of cytosine residues with the help of DNA methyltransferases (DNMTs) by creating 5-mC (5-methyl cytosine). Correct CpG methylation plays a major role in X-chromosome inactivation and the stability of chromatin and is directly associated with fertility potential and early embryo development. On the other hand, defective DNA methylation is characterized by unstable CpG methylation patterns, which correlate with sperm movement as well as DNA and chromatin integrity and lead to infertility or poor semen quality [74,75,76,77,78].

As an elementary unit of chromatin, the nucleosome structure consists of small DNA fragments wrapped around the histone octamer including a pair of H2A, H2B, H3, and H4 histones. Post-translational histone modification is characterized as a covalent transformation of the N-terminal lysine-rich tails, which is enzymatically regulated by acetyltransferases, deacetyltransferases, methyltransferases, and demethylases, especially in the H3 and H4 histones. In spermatozoa, the most often histone modifications involve acetylation, methylation, and phosphorylation as well as ubiquitination, glycosylation, sumoylation, crotonylation, ADP-ribosylation, propionylation, and butyrylation [73].

The process of histone post-translational modifications is involved in the regulation of spermatogenesis, transcription, and DNA repair and replication as well as the condensation of chromosomes. During spermatogenesis, the majority (85–95%) of histones is excluded and the remaining (5–15%) histones are replaced by small arginine-rich sperm-related nuclear proteins called protamines, which are incorporated into the chromatin and initiate DNA condensation in the sperm head [79,80,81]. The remodelation of chromatin is an ATP-dependent mechanism, which includes the relocation of nucleosomes and structural changes that start with the redistribution of the chromatin structure with the help of SWI/SNF, ISW1, and MI-2 chromatin remodeling protein complexes, which can be used as potential markers of postfreezing transcriptional activity in mammalian spermatozoa [82]. 

A member of the JHDM (Jmjc domain-containing histone) family Jhdm2a plays a key role in the methylation of the H3K9 histone and regulation of gene silencing or cell growth. In a study by Zeng et al., the gene as well as protein expression of selected epigenetic-related genes including Jhdm2a and Kat8 decreased due to cryopreservation of boar spermatozoa. The insufficiency of Jhdm2a leads to inappropriate methylation of transition nuclear proteins, which may cause male infertility since the expression of Jhdm2a positively correlated with postmeiotic chromatin condensation. On the other hand, the upregulation of Jhdm2a induced methylation of the target protamine 1 promoter and increased gene silencing and chromatin condensation, which inhibited sperm motility of boars. Acetylation of H4K16 is thought to ubiquitously express the Kat8 or MYST1 gene during spermatogenesis. When Kat8 is downregulated, it may cause apoptotic changes in mature boar spermatozoa and affect their motility or viability as well as early embryonic development in mice [83,84]. 

According to Salehi et al. [85], epigenetic patterns of cryopreserved rooster spermatozoa were changed after thawing. There were no differences in DNA methylation, but a significant reduction in H3K9 acetylation and H3K4 methylation was observed between two experimental groups frozen in different extenders when compared to the untreated fresh group. Interestingly, the level of DNA fragmentation was increased in groups treated with extenders, which negatively correlated with DNA methylation, which decreased in both treated cryopreserved groups compared to the control. In contrast, in stallions, the cytosine methylation of sperm DNA statistically increased after cryopreservation, and extenders had no effect on the level of epigenetic alternations [86]. 

In the case of human spermatozoa, a heterogenous presence of histone methylation on H3K4Me1, H3K9Me2, H3K4Me3, H3K79Me2, and H3K36Me3 was detected in mature cells with poor morphology and decreased functional quality, which makes them a potential marker for the prediction of freezability. However, the presence of histone acetylation H3K4Ac and H4K5Ac was proved in neither normal nor abnormal mature human spermatozoa [87]. An abnormal DNA methylation status of specific genes such as MEST (mesoderm-specific transcript), H19 (H19 imprinted gene), and MTHFR (methylenetetrahydrofolate reductase) is connected to male infertility. In some patients, cryopreservation can promote epigenetic alternations and activate alternative splicing events or compromise the functional activity of mitochondria [70,88]. 

Another approach to studying epigenetic modifications is through noncoding RNAs, particularly microRNAs (miRNAs), which have the ability to regulate the microepigenetic expression pattern of various genes at the post-transcriptional level, while dysregulation of miRNAs may lead to reproductive failure and male infertility [70,89].

## 6. Protein Markers

Recent technological advances have enabled the scientific community to shift their attention towards investigating the molecular factors associated with the seminal proteome that could predict the resilience of male gametes towards low temperatures and thus affect the fertilization process of frozen–thawed semen samples [30]. In fact, pivotal comprehensive proteomic studies have unraveled an exceptional diversity of the protein constellation in the seminal plasma and spermatozoa that has become a base for the assessment of semen quality characteristics, in particular for the prediction of the fertility potential of spermatozoa [90,91,92]. At the same time, high-throughput mass spectrometry demonstrates that several proteins and peptides could become useful markers for sperm function in fresh as well as cryopreserved semen [93]. As such, it has been suggested that proteomic profiling could assist in a more accurate estimation of the cryoresistance or cryosensitivity of semen, which is known for its inter- as well as intraspecies variability [30]. 

Proteomic profiling of semen is a fairly complicated process, primarily because the ejaculate is a complex fluid consisting of two major components—the seminal plasma and spermatozoa. Ejaculated spermatozoa contain their own unique proteomes localized in different subcellular structures, which may be differently associated with sperm freezability [94]. At the same time, male gametes undergo several physiological and functional modifications following ejaculation that are affected by several factors, particularly by the seminal plasma proteins that are synthesized in the epididymis and accessory sexual glands during ejaculation [95]. As such, it is feasible to assume that the seminal plasma proteome may equally contribute to the resulting sperm freezability. For the purposes of this review, we will address the most promising candidates for the prediction of post-thaw semen quality according to their localization in the seminal plasma or spermatozoa.

### 6.1. Sperm Proteins

The protein constellation in spermatozoa and its respective compartments has been well documented using different proteomic-based techniques in a plethora of mammalian species [90,96,97,98]. Accordingly, differential expression patterns of certain families or types of sperm proteins following cryopreservation have been studied to estimate semen freezability [99,100,101,102,103]. Nevertheless, as emphasized by Fraser [57], while several sperm-specific proteins have been suggested to act as markers of sperm cryotolerance [28,32,99,104], their dynamics by and large depend on the presence of their corresponding mRNAs that shall be eventually translated if and when needed. Different proteins have been reported to be up- or downregulated in samples of varying degrees of post-thaw quality, most of which play essential roles in the energy metabolism of spermatozoa such as glycolysis, citric acid cycle, and oxidative phosphorylation as well as cell motility, protein biosynthesis and proteolysis, protein transport, and response to oxidative stress. Nevertheless, out of these proteins, several have been streamlined as possible hallmarks of sperm cryoresistance because of their persistent reappearance as being underexpressed or overexpressed in frozen–thawed spermatozoa collected from different species. 

Mammalian heat shock proteins (HSPs) are highly conserved molecular chaperones, which are induced in cells exposed to high temperatures, viral infection, drugs, and chemical or physical stress, where they act as cytoprotective agents through the maintenance of protein homeostasis and prevention of caspase-triggered cell death [105,106]. Activated HSPs assist in the refolding of denatured proteins, thus preventing the eventual accumulation of misfolded proteins and subsequent proteotoxic cell death [106,107]. Several HSP family members have been reported to be expressed in the testis and spermatozoa of mice, rats, boars, bulls, and humans [105,106,108]. Among these, HSP90 has been primarily localized in the sperm tail [105] and suggested to play a role in mediating sperm capacitation, hyperactivation, and fertilization [109]. Numerous proteins, such as tyrosine kinases and serine–threonine kinases, have been associated with HSP90 activity and its impact on sperm motion [110]. In the meantime, HSP70 has been shown to relocalize from the acrosome to the equatorial segment, postacrosomal region, and midpiece during sperm capacitation in bulls [111]. Furthermore, Spinaci et al. [112] reported that HSP70 is relocalized and translocated from the inner to the outer plasmalemma leaflet following acrosome reaction in boars. The expression patterns of both HSPs have been reported to be proportional to the post-thaw sperm motility in bulls [102,103]. According to Yeste [32], HSP70 levels were found to be able to predict sperm cryoresistance in boars. In another study, it was demonstrated that greater levels of HSP90 in high-freezability boars might confer increased sperm tolerance to low temperatures [113]. Furthermore, greater expression levels of HSP90 were detected in bovine spermatozoa with high cryoresistance and motion kinetics, indicating that the protein can be used as a marker for semen freezability [114]. Accordingly, recent studies have suggested that HSP supplementation to freezing extenders could protect spermatozoa against cryo-induced motility inhibition and cell death [115].

A-kinase anchor proteins (AKAPs) and their precursors (pro-AKAPs) are highly conserved major components of the fibrous sheath located in the sperm flagellum. Their primary function lies in the proper anchoring of cAMP-dependent kinases (most commonly protein kinase A-PKA) to specific subcellular regions via the regulatory subunits of the enzymes. By such action, AKAPs increase the specificity of kinases by directing them towards subcellular targets, which is a prerequisite for proper kinase signaling [116]. In male gametes, AKAP4 and pro-AKAP4 have been reported to anchor PKA to the fibrous sheath, hence placing the enzyme in close proximity to target proteins in the flagellum. A properly activated PKA then phosphorylates serine and threonine residues, activating downstream targets that are primarily located in the flagellum. As such, it has been suggested that AKAP4-mediated targeting of PKA may be important for the organization of flagellar PKA signaling and sperm motility [117,118]. According to Turner et al. [118], a decrease in equine sperm motility following cooling or freezing could be partially caused by the decreased ability of AKAP4 to properly bind to the regulatory subunit of PKA. Furthermore, it has been reported that an increase in the expression of AKAPs in frozen–thawed spermatozoa might be associated with their capacitation patterns [99]. In boar sperm lysates, the levels of particularly AKAP3 and AKAP4 were associated with semen freezability [32,99]. Finally, Blommaert et al. [119] suggest that AKAP4 and pro-AKAP4 concentrations could become promising biomarkers of fresh and frozen–thawed sperm quality in stallions.

Aquaporins (AQPs) are ubiquitous transmembrane proteins, which are amongst the best-studied indicators of sperm cryotolerance. Thirteen AQPs have been identified in mammalian spermatozoa and their primary roles lie in regulating the passage of water and minor molecular solutes such as glycerin between the sperm cell and the semen extender [120,121]. While AQP3, AQP7, and AQP11 have been most frequently associated with cryoresistance and the fertilizing ability of frozen–thawed spermatozoa in boars [121,122], stallions [123], and bulls [124], their exact function and mechanism of action differ greatly among species. AQP7 is considered a universal regulator of motility and membrane lipid architecture during sperm cryopreservation [122,123] and could be considered a predictor of sperm resilience, while AQP3 activity has been linked to cryotolerance exclusively in boars [125,126]. On the other hand, AQP11 has been reported to be engaged in the cryotolerance and post-thaw fertilizing ability only in the case of bull spermatozoa [124,127]. As such, their species-specific involvement in sperm resistance to low temperatures needs to be elucidated further.

In a recent report, Song et al. [128] observed that cytosolic 5-nucleotidase 1B (NT5C1B) associated with the regulation of nucleotide metabolism and fumarate hydratase (FH) involved in the tricarboxylic acid cycle were abundantly expressed in bovine spermatozoa highly tolerant to cytoprotective agents with potentially deleterious effects on male gametes. In the meantime, Casas et al. [129] suggested that glucose transporter 3 (GLUT3) involved in energy provision for protein phosphorylation from glucose substrates could act as a predictor of good sperm freezability in boars. According to other studies on boar spermatozoa [32,99], the levels of outer dense fiber 2 (ODF2), supporting and protecting the sperm tail during epididymal transit and ejaculation [130]; voltage-dependent anion channel 2 (VDAC2) considered the main pathway for metabolite diffusion across the mitochondrial outer membrane [131]; and acrosin-binding protein (ACRBP) that maintains proacrosin as an enzymatically inactive zymogen in the acrosome [132] were associated with semen freezability. 

Finally, proteomic profiling of frozen–thawed human spermatozoa [101] unraveled a significant upregulation of several proteins including clusterin (CLU) as a major sperm glycoprotein [133], tektin (TEKT) 2 and 3 present in the axoneme and periaxonemal structures necessary for progressive motility of spermatozoa [134], and L-xylulose reductase (DCXR), a multifunctional protein involved in various enzymatic and protein interaction processes [135]. On the other hand, proteins such as acrosin (ACR), the major proteinase present in the acrosome of mature spermatozoa [136], calmodulin (CALM1) involved in calcium signal transduction pathways [137], cytochrome (CYC2), and NADH-cytochrome b5 reductase2 (CYB5R2) as critical elements involved in sperm mitochondrial biogenesis and metabolism [138,139] were significantly decreased following cryopreservation, resulting in reduced post-thaw semen quality. A summary of the most studied sperm protein markers associated with sperm freezability is provided in Table 2.

### 6.2. Seminal Plasma Proteins

Seminal plasma, which accounts for 95% of the ejaculate, plays essential roles in the natural fertilization process since it contributes to timely sperm capacitation, hyperactivation, acrosome reaction, and sperm-oocyte fusion [140]. Its components offer nourishment and protection to male gametes throughout their journey in the male and female reproductive tract [141]. In the meantime, tissue-specific proteins present in the seminal plasma may serve as potential biomarkers for the assessment of semen quality or diagnosis of male reproductive pathologies [142,143]. The evolution of high throughput proteomic tools has greatly enabled the evolution in the understanding of the seminal plasma proteome, which may become a valuable source of markers that could predict sperm cryotolerance or cryosensitivity. 

Osteopontin (OSP) is a multifunctional acidic glycoprotein that is involved in various physiological processes, including bone remodeling, immune regulation, inflammation, and fertilization [144]. These roles are attributed to the ability of OSP to interact with membrane proteins, specifically with integrins located on the surface of male and female gametes, which regulate cell adhesion, cell–cell interactions, tissue, and extracellular remodeling [145]. It has been previously reported that OSP modulates capacitation, promotes acrosome reaction, and inhibits sperm apoptosis [146,147]. Moreover, in vitro treatment of frozen bovine, buffalo, or porcine oocytes or spermatozoa with OSP has been shown to increase fertilization rates, cleavage, and embryo development [148,149,150]. As such, seminal plasma OSP has been regarded as a marker of bovine fertility [146,151], and its overexpression has been observed in semen samples with a good post-thaw sperm quality of Guzerat [152], Zebu [153], and Holstein bulls [154].

Binder of sperm (BSP) proteins are relatively small acidic proteins found in a multitude of mammals including bulls, stallions, rams, boars, bucks, humans, rats, mice, and rabbits (reviewed by [155]). It is believed that BSPs bind to the outer leaflet of sperm membranes during ejaculation. Besides playing important roles as mediators of capacitation [156], it has been reported that BSPs act as chaperones in sperm motility and viability and mediate sperm-egg interactions [157]. Moreover, BSPs have been implicated in sperm freezability due to their ability to bind to egg yolk low-density lipoproteins [158] and milk components [159] of semen extenders, thus protecting spermatozoa exposed to low temperatures from the loss of membrane lipids. Out of the BSP superfamily, BSP1 and BSP5 have been detected in the seminal plasma of bulls with high sperm freezability [100,154].

Clusterin (CLU) is a small glycoprotein found in the seminal plasma and sperm surface of several species including dogs [160], bulls [152], boars [161], and rams [162]. The protein is involved in sperm maturation, cell adhesion, and lipid transport [163]. Particularly in bulls, CLU levels have been inversely correlated to morphologically normal spermatozoa, suggesting that the protein selectively binds to male gametes with morphologic defects [164]. Recently, Jobim et al. [153] have unraveled its high expression in bovine semen samples with high freezability, proposing that CLU could act in a similar manner to small heat shock proteins during the freeze–thaw process by binding unfolded proteins and blocking their aggregation, which may be particularly important in the prevention of DNA oxidation and subsequent sperm damage [165]. As such, Gomes et al. [154] have speculated that CLU could act as a member of a selected group of components defined as “seminal plasma sperm-protecting molecules” that includes proteins such as albumin, superoxide dismutase, catalase, glutathione peroxidase, and peroxiredoxins.

Fibronectin-1 (FN-1) is a high-molecular-weight glycoprotein found in the plasma, extracellular matrix, and on the cell surface. Pivotal studies in humans have unraveled that FN-1 promotes sperm maturation, capacitation, and sperm–egg interactions [166] through the cyclic adenosine monophosphate and protein kinase pathways [167]. In boars, FN-1 is one of the most abundant proteins found in the seminal plasma [168], which has been found to be negatively associated with the proportion of sperm tail abnormalities [161]. Rungruangsak et al. [169] have observed significant fluctuations of FN-1 in boar semen according to their freezability, with the highest levels of the protein found in good freezers, suggesting that the protein could become a potential freezability marker for boar semen cryopreservation. This finding was supported by Vilagran et al. [170], according to whom FN-1 could stabilize boar sperm activity and provide defense to spermatozoa during the freeze–thaw process. 

The cysteine-rich secretory protein-3 (CRISP-3) genotype is present in large amounts in stallion seminal plasma [171] and has been positively associated with the first-estrous cycle conception rate of mares subsequent to parturition as well as stallion sperm quality [171,172]. At the same time, CRISP-3 was shown to be a possible seminal plasma marker for the identification and selection of stallions with optimal semen freezability [173,174]. Accordingly, CRISP-3 was more abundant in samples from stallions with greater semen freezability. Recently, it was also speculated that CRISP-3 might be a potential seminal plasma marker for greater semen freezability in donkeys [175].

In the meantime, horse seminal plasma protein 2 (HSP-2) was shown to be a possible seminal plasma marker of increased semen freezability in a study conducted by Jobim et al. [174] who evaluated protein profiles of seminal plasma from stallions with varying semen freezability profiles. The precise mechanism of the protein is still unknown; however, it has been suggested that HSP-2 may provide better protection to the sperm membrane architecture and properties during the freezing procedure.

According to Wysocki et al. [176], the presence and activity of N-acetyl-β-hexosaminidase (β-HEX) in the seminal plasma could also act as a reliable indicator of boar sperm cryosensitivity. β-HEXs are found on the sperm surface and in the acrosome and act as lysosomal enzymes that hydrolyze acetylglucosamine and acetylgalactosamine residues from oligosaccharide residues in glycoconjugates [177]. The enzyme is capable of modulating the binding properties of the plasma membrane to zinc, thus decreasing its resistance to the cryopreservation process. Since the activity of β-HEXs has been found in samples with low post-thaw membrane integrity and more intense lipid peroxidation, this enzyme may be suitable to predict low freezability potential in boars. Similarly, the Niemann-Pick C2 protein (NPC2), which is related to cholesterol efflux from the sperm membrane, could be associated with low cryotolerance of boar spermatozoa. A significantly enhanced expression of the protein was found in semen with poor freezability ejaculates by Valencia et al. [178], suggesting that NPC2 would impair the sperm membrane fluidity by contributing to lipid-phase separations and imbalance in the transmembrane transport of calcium [33].

Inversely, lipocalin-type prostaglandin D synthase (L-PGDS), known to act as a carrier of retinoids such as retinoic acid and retinol [179], which modify the permeability of the plasma membrane while interacting with phospholipids, has been observed in boar semen of superior freezability [180]. This phenomenon may be related to the ability of L-PGDS to stabilize the sperm membranes and prevent cryocapacitation. L-PGDS has been also encountered in bovine seminal plasma although with conflicting results. While according to Jobim et al. [153], seminal plasma samples from bulls of low freezability presented the spot corresponding to L-PGDS with optical density significantly higher than samples from bulls with high semen freezability, Roncoletta et al. [181] found 40% more of this protein in bull seminal plasma with better freezability and attributed this to L-PGDS’s capacity to participate in modifications of cell membrane permeability. As such, the potential of L-PGDS to predict sperm freezability in bulls is subject to further studies.

Finally, spermadhesins have been found to be the second most abundant protein family detected in the seminal fluid of bulls presenting with a superior post-thaw sperm quality by Gomes et al. [154]. Like BSPs, spermadhesins are found in bulls [152], buffaloes [182], rams [162], and boars [161], and previous reports indicate that these may affect sperm membrane stability and capacitation, sperm–oviduct interactions, and sperm–egg binding [183].

A new perspective on the prediction of sperm freezability in boars has been provided by Barranco et al. [184] who revealed that numerous cytokines present in the seminal plasma could be predictive of the outcome of boar sperm cryopreservation. Their experimental design based on multiple linear regression models, with a Bayesian approach for variable selection, revealed that the anti-inflammatory interferon gamma (IFN-γ) was included in the models predicting changes in all sperm attributes for cryopreserved semen. Boar spermatozoa have receptors to IFN-γ [185] and its concentration is high in boar seminal plasma. Moreover, IFN-γ may activate several other cytokines [186], which may affect sperm cryosurvival in terms of total and progressive motility, viability, and lipid reoxidation. Whilst this study may be considered pivotal, it establishes a new ground for the understanding of the roles that immunity may play during the freeze–thaw process. A summary of the most studied seminal plasma proteins associated with sperm freezability is provided in Table 3.

## 7. Fatty Acids

While the physiological roles of fatty acids as energy sources and structural components of cells are well known, cryopreservation-induced changes to their structure, function, or composition have been not completely elucidated to date. Pivotal studies have reported that lauric, myristic, and oleic acids were two-fold more abundant in spermatozoa collected from stallions considered good freezers [187]. Furthermore, according to Vieira et al. [188], spermatozoa with large amounts of docosahexaenoic acid (DHA) and stearic acid presented with superior post-thaw motility. However, the specific mechanisms underlying the role of fatty acids in sperm cryotolerance remain by and large unknown, even though cells with a high proportion of PUFAs have a higher degree of membrane fluidity and flexibility, which is provided by an abundance of double bonds present in the fatty acid chains [189].

## 8. Strategies to Improve the Semen Quality of Poor Freezers

For several decades now, scientists have attempted to reduce the detrimental effects of cryopreservation on spermatozoa. The current approach is based on so-called defensive strategies, in which different supplements are added to freezing media in order to offer protection to male gametes against cryodamage. Cryoprotective agents [190,191], antioxidants [192], antifreeze proteins [193], fatty acids [194], and plant essential oils [195] are amongst the most studied ones. As critically reviewed by Zini and Al-Hathal [192], the outcomes of cryosupplementation are often conflicting. Some reports have shown that several antioxidants, proteins, and fatty acids are effective in reducing ROS levels and in preventing the decline in sperm motility during processing [196,197,198,199]. In contrast, other studies have reported that alternative molecules with cryoprotective properties were ineffective in protecting spermatozoa from the loss of motility during processing and storage [200,201,202]. Moreover, antioxidants and cytoprotective molecules appear to be of limited value in protecting sperm DNA during freezing [203]. In some cases, antioxidant supplementation in vitro may even cause excessive DNA damage, while inadequate doses of cytoprotectants are per se toxic to the sperm cell [202,204]. In this sense, a general consensus among animal and human andrologists has emerged calling out to optimize the protocols for semen storage by increasing the sperm life span without compromising the function of male gametes. This would translate into increased fertilization rates and assisted reproduction outcomes. 

One thing all previous studies on cryosupplements have in common is a generic approach. The samples are generally treated according to inclusion/exclusion criteria, which are normally based on the initial sperm motility, without taking other individual properties of semen into consideration. As such, even when complying with the preestablished criteria for sample inclusion in a study, some individuals may respond to a specific treatment better than others, leading to often contradictory or inconsistent outcomes. Sometimes, targeting antioxidant supplementation may better confront oxidative stress during cryopreservation and lead to improved post-thawed quality than untargeted antioxidants [205]. Only the combination of adjusted cryopreservation conditions and final glycerol concentration may significantly improve cryotolerance in ejaculates of suboptimal sperm freezability [206]. A simple and cost-effective solution may bring supplementation of the freezing extender with the seminal plasma of good freezers. Such a solution may improve sperm motility, acrosome integrity, inhibit capacitation-like changes, and increase cryoresistance and oxidative balance [207,208]. Another opportunity to improve the freezability of ejaculates lies in changes in dietary modifications. A highly energetic diet, particularly in the long-term horizon, can lead to deteriorated sperm quality and freezability. On the other hand, the consumption of dietary fats, microminerals, and vitamins may improve sperm quality and thus semen freezability [209]. A new insight into the improvement of post-thaw sperm quality brings density gradient centrifugation before cryopreservation. In particular, creating a high-quality genetic reserve for rare species is more valuable than a higher total sperm concentration [210]. A nanotechnology-based approach may also bring unique accuracy to this field. Durfey et al. [211] published their study based on nanoselection using biocompatible magnetic nanoparticles to target Annexin-V-positive and lectin (acrosome-reacted)-positive spermatozoa. Such magnetic nanoselection represents an auspicious tool for improving cryotolerance. However, further research is needed to make this protocol friendly and acceptable for the common cryopreservation process. 

Taking all arguments together, the ultimate goal of the future lies in a greater elimination of the intrinsic and extrinsic factors that may endanger sperm survival at low temperatures before the freezing process and to do so with the selection of appropriate sample processing protocols, freezing techniques, and cryosupplements that are most feasible for the individual characteristics of the sperm sample in order to obtain a high proportion of viable spermatozoa following the freeze–thaw process.

## 9. Conclusions

The search for new freezability markers using transcriptomics, bioinformatics, and proteomics enabling the characterization of molecular changes in spermatozoa prior to, during, and following cryopreservation may offer new perspectives to enhance marker-assisted sperm selection programs in human as well as animal andrology. At the same time, such freezability markers may also help to unravel the biological functions of sperm-derived markers in the mechanism underlying cryotolerance of male gametes, which may have a significant impact on the technology of semen cryopreservation. Summarizing the evidence collected from the currently available literature, we may suggest that acrosin binding protein, osteopontin, aquaporins, and heat shock proteins have been highlighted as potential biomarkers that could predict sperm resistance to cryopreservation in animals. In the meantime, clusterin, acrosin, and protamines are possible hallmarks of human sperm quality before freezing. Nevertheless, their potential as predictors of sperm cryotolerance needs to be verified in large-scale multicenter studies. 

## Figures and Tables

**Table 1:** Genetic markers associated with the freezability of mammalian spermatozoa.

Biomarker	Source	Fertility Association	Regulatory Function	Reference
TSSK6, PRM 1, PRM 2	Bulls	Decreased in poor freezers	(TSSK6) DNA condensation during post-meiotic chromatin remodeling; (PRM1/2) packaging and protecting DNA	[55]
PLCZ1, CRISP2, CLGN1, ADAM5P	Bulls	Expressed in high post-thawed motile spermatozoa	(PLCZ1 and CRISP2) regulation of ion channels and Ca^2+^ oscillations during sperm capacitation; (CLGN1) spermatogenesis regulator; (ADAM5P) sperm–egg fusion	[56]
RLP31, GCLC, CYB5R4, CCT5	Bulls	Upregulated in good freezers	(RLP31) protein interactions, (GCLC) catalyzes the ATP-dependent ligation; (CYB5R4) endoplasmatic reticulum stress response pathway; (CCT5) folding of proteins upon ATP hydrolysis	[57,58,59]
COX7C	Bulls	Downregulated in low-fertility group	(COX7C) catalysis of electron transfer from reduced cytochrome c to oxygen	[60]
HSPD1	Boars	Expressed in good-freezability group	(HSPD1) protection against heat shock	[61]
TXNRD1, HSPA4L, ATP1B1	Boars	Associated with poor/reduced freezability	(TXNRD1) inflammation and oxidative stress regulator; (HSPA4L) protection against heat stress; (ATP1B1) Na^+^/K^+^ transmembrane exchange	[62]
FOS, NFATC3, EAF2, BAMBI, PTPRU, PTPN2, MT-ND, ACADM	Boars	Expressed in poor freezers	(FOS) glucose metabolism; (NFATC3) inflammatory regulator; (EAF2) DNA repair mechanisms; (BAMBI) sperm cell differentiation; (PTPRU and PTPN2) signaling molecules; (MT-ND) NADH dehydrogenase synthesis; (ACADM) acyl-CoA dehydrogenase synthesis	[59]
TPX-1, LDHC, AKAP4, HSBP1, CLU	Humans	Overexpressed in samples with low fragmentation index	(TPX-1) catalyzes the reduction of hydrogen peroxide; (LDHC) maintenance of glycolysis and ATP production in sperm flagellum, (AKAP4) regulator of sperm motility; (HSBP1) protection against heat stress; (CLU) sperm maturation and capacitation	[63]
ACO2, PKM2	Humans	Present in good freezers	(ACO2) mitochondrial metabolism; (PKM2) generation of ATP	[64]

**Table 2:** Sperm protein markers associated with freezability of mammalian spermatozoa.

Biomarker	Source	Fertility Association	Reference
A-kinase anchor protein 4	Stallions	Decreased levels proportional to a decline in post-thaw spermatozoa motility	[118]
		High expression in fresh and frozen–thawed spermatozoa of good freezers	[119]
	Boars	Overexpression in samples with high freezability	[32]
Acrosin	Humans	Underexpressed in frozen–thawed spermatozoa in comparison to native spermatozoa	[101]
Acrosin-binding protein	Boars	Higher levels in good freezers in comparison to poor freezers	[132]
Apoptosis-inducing factor 1-mitochondrial	Humans	Underexpressed in frozen–thawed spermatozoa in comparison to native sperm	[101]
Aquaporin 3	Boars	High amounts in spermatozoa of good freezability	[122,125]
	Stallions	High amounts in spermatozoa of good post-thaw quality	[123]
	Bulls	High amounts in spermatozoa of good post-thaw quality	[124]
Aquaporin 7	Boars	High amounts in spermatozoa of good freezability	[122,125,126]
	Stallions	High amounts in spermatozoa of good post-thaw quality	[123]
Aquaporin 11	Bulls	High amounts in spermatozoa of good post-thaw quality	[124,127]
	Stallions	High amounts in spermatozoa of good post-thaw quality	[123]
ATP1β1	Bulls	Significantly higher in spermatozoa highly tolerant to cryopreservation	[30]
Calmodulin	Humans	Underexpressed in frozen–thawed spermatozoa in comparison to native sperm	[101]
Carbonic anhydrase 2	Humans	Underexpressed in frozen–thawed spermatozoa in comparison to native sperm	[101]
Clusterin	Humans	Overexpressed in frozen–thawed spermatozoa in comparison to native spermatozoa	[101]
Cytochrome	Humans	Underexpressed in frozen–thawed spermatozoa in comparison to native spermatozoa	[101]
Cytosolic 5-nucleotidase 1B	Bulls	Overexpressed in high cryoprotective agent-tolerant spermatozoa	[128]
Fumarate hydratase	Bulls	Overexpressed in high cryoprotective agent-tolerant spermatozoa	[128]
Glucose transporter 3	Boars	High in good-freezability spermatozoa	[129]
Heat shock protein 70	Bull	Expression proportional to frozen–thawed spermatozoa motility	[103]
	Boar	Overexpression in high-freezability boars	[32,113]
Heat shock protein 90	Bull	Expression proportional to frozen–thawed spermatozoa motility	[102]
		Higher levels in bulls with high cryoresistance	[104]
	Boars	High in good-freezability spermatozoa	[129]
Histone H4	Humans	Overexpressed in frozen–thawed spermatozoa in comparison to native spermatozoa	[101]
Inositol monophosphatase 1	Humans	Underexpressed in frozen–thawed spermatozoa in comparison to native spermatozoa	[101]
L-xylulose reductase	Humans	Overexpressed in frozen–thawed spermatozoa in comparison to native spermatozoa	[101]
NADH-cytochrome b5 reductase2	Humans	Underexpressed in frozen–thawed spermatozoa in comparison to native spermatozoa	[101]
Outer dense fiber 2	Boars	Increased in frozen–thawed spermatozoa	[99]
Phosphoglycerate mutase 2	Humans	Underexpressed in frozen–thawed spermatozoa in comparison to native spermatozoa	[101]
Triosephosphate isomerase 1	Boars	Increased in frozen–thawed spermatozoa	[99]
Voltage-dependent anion channel 2	Boars	Higher levels in good freezers in comparison to poor freezers	[131]

**Table 3:** Seminal plasma protein markers associated with freezability in mammalian spermatozoa.

Biomarker	Source	Fertility Association	Reference
Binder of sperm proteins	Bull	High in samples with good post-thaw quality	[154]
		Abundant in seminal plasma of high semen freezability	[100]
Clusterin	Bull	Abundant in seminal plasma of high semen freezability	[153]
		High in samples with good post-thaw quality	[154]
Cysteine-rich secretory protein-3	Stallion	High in samples with good post-thaw quality	[173]
		Abundant in seminal plasma of high semen freezability	[174]
	Donkey	High abundance in semen with optimal freezability	[175]
Fibronectin-1	Boar	High abundance in good-freezability semen	[169,170]
Horse seminal plasma protein 2	Stallion	Abundant in seminal plasma of high semen freezability	[174]
Lipocalin-type prostaglandin D synthase	Boar	Abundant in seminal plasma of high semen freezability	[180]
N-acetyl-β-hexosaminidase	Boar	High activity in semen with low post-thaw quality	[176]
Niemann-Pick C2 protein	Boar	High abundance in semen with low post-thaw quality	[178,180]
Osteopontin	Bull	Abundant in seminal plasma of high semen freezability	[152,153]
		High in samples with good post-thaw quality	[154]
Spermadhesins	Bull	High in samples with good post-thaw quality	[154]

## Data Availability

Data collected for the purposes of this paper are available upon reasonable request from the corresponding author.

## Supplementary Materials

The following supporting information can be downloaded at: https://www.mdpi.com/article/10.3390/ijms24043379/s1.
